# Supplementary material for: “I just felt that everything came tumbling down around me”—Barriers in cancer care for patients with severe mental illness: A qualitative study
Source: PLoS One. 2025 Jan 27;20(1):e0314313. doi: 10.1371/journal.pone.0314313 (PMC11771855; doi:10.1371/journal.pone.0314313)
Supplement: S1 Table — (DOCX) [file pone.0314313.s001.docx]

**Supporting Information**

**S1 Table. Interview guide**

| Research questions | Interview questions |
| --- | --- |
| What is the experience of being a patient with both a psychiatric and a cancer diagnosis with treatment trajectories in both settings? | You are a patient both at the Department of Oncology and in the psychiatric setting. What is that like?  How do the staff in the psychiatric setting deal with your cancer diagnosis?  How do the staff at the oncology department deal with your psychiatric diagnosis? |
| What is the role of general practice and the municipality in the cancer trajectory? | How is the contact between you and your general practitioner?  Are you connected to the municipality? If so, how is your contact with them? |
| Which barriers and facilitators do the patients see in terms of having the most suited cancer treatment? | What is the cancer trajectory like for you?  What is difficult in the trajectory?  What works well?  If you could decide how things were done, what would this cancer trajectory have been like?  What helps you through the cancer trajectory? |
