# Supplementary material for: “I just felt that everything came tumbling down around me”—Barriers in cancer care for patients with severe mental illness: A qualitative study
Source: PLoS One. 2025 Jan 27;20(1):e0314313. doi: 10.1371/journal.pone.0314313 (PMC11771855; doi:10.1371/journal.pone.0314313)
Supplement: Attachment — Submitted filename: Rebuttal letter.docx [file pone.0314313.s002.docx]

**Rebuttal letter**

*Author comments are in italics.*

**PONE-D-24-23981**

**”I just felt that everything came tumbling down around me” - Barriers in cancer care for patients with severe mental illness: A qualitative study**

**PLOS ONE**

*Dear Editor,

We appreciate the opportunity to respond to the comments provided by the academic editor and the two reviewers. Below, we have addressed each point raised, providing detailed responses to the questions and suggestions. We are grateful for the reviewers' insightful feedback, which has significantly contributed to the improvement of our manuscript.*

*We hope that the revised manuscript now meets the criteria for publication in PLOS ONE. Please let us know if any additional changes are required.*

*On behalf of all the authors,*

*Lars Henrik Jensen*

==============================

**ACADEMIC EDITOR:**

This manuscript has merit and would be of great interest to readers. There are few areas that require further clarification before it can be considered for acceptance:

- Reviewer 2 has queried regarding the 'hermeneutical approach' employed in this manuscript. It would be advisable to explicitly state the characteristics of hermeneutical approach in the methods. I do note that the manuscript has reported the application of reflexivity and interpretive analysis. Rephrasing the methods to be more explicit regarding the hermeneutical approach may be helpful.

*We appreciate the comment and have revised the Methods section accordingly. Please refer to lines 94-104. Additionally, we have included two references from Gadamer and Lindseth. Please also see the comment to Reviewer 2 below.*

- The definition of severe mental illness was provided in lines 97-99. As mentioned by Reviewer 1, the application of this definition of severe mental illness would require clarification. The participants of the study consisted of patients with mood disorder, anxiety disorder and one patient with psychotic disorder. All these could have a range of mild to severe illness, as patients with severe mood disorder may also have psychotic symptoms. Elaboration of the severity of these diagnoses may help to justify the definition for severe mental illness.

*We recognize that the reviewer’s comments indicate some uncertainty regarding the distinction between "severe mental illness" and "mental illness" within the context of our study. It has become clear that there is some complexity in how these terms are applied. The references cited in our manuscript are partially derived from studies involving patients with SMI, which typically refers to individuals with diagnoses such as schizophrenia and affective disorders. The reviewer is correct in pointing out that our study also includes other diagnostic groups, which may contribute to the confusion, particularly given the presence of multiple diagnoses among participants. This raises questions about whether these are primary and secondary diagnoses, and if so, it indeed becomes challenging to understand the exact composition of our participant group. It is crucial to clarify that all participating patients were recruited from secondary psychiatric services, meaning they were either inpatients or receiving outpatient treatment. Therefore, they all have a history of prolonged and complex engagement with psychiatric care. After the WHO definition, we have added ‘In line with this definition, participants in this study were recruited from secondary psychiatric services, including both inpatient and outpatient settings, with the additional criterion that they had a history of prolonged and complex engagement with psychiatric care.’ (line 117)*

- Additional quotes to support the interpretation of results as mentioned by Reviewer 1 is required.

*Please see below, Reviewer 1.*

- In line with hermeneutical approach, providing more context would be helpful. For example, which patients had undergone ECT? What cancer phase were the participants in at the time of the study? Had they undergone surgery, chemotherapy, or radiotherapy?

*Thank you for the feedback. We have provided additional context to align with the hermeneutical approach. Specifically, we have detailed which patients had undergone ECT, clarified the cancer phases of the participants at the time of the study, and outlined their prior treatments, including surgery, chemotherapy, or radiotherapy. This will enhance understanding of the participants' treatment trajectories and overall experiences.*

*In ‘Participants’:*

*“During the study, three patients were undergoing curative or adjuvant oncology treatment, while one patient was receiving life-prolonging medical oncology treatment. Another patient, who had been discontinued from adjuvant oncology treatment, was instead being monitored with control CT scans at the time of their inclusion in the study. This patient was still considered able to provide valuable information regarding the treatment trajectory and was therefore retained in the study.
Three patients were undergoing radiotherapy, and three patients (with two overlapping with radiotherapy) were receiving medical oncology treatments, administered either as injections or oral tablets. Additionally, three patients had previously undergone surgical removal of their cancer, and two had received intravenous chemotherapy before the study began.
Two patients were undergoing electroconvulsive treatment (ECT), and all patients were receiving psychopharmacological treatment.”*

- Why did the study specifically include the role of GPs as it was not evident in the objectives?

*Thank you for raising this point. We recognize that the initial manuscript did not provide sufficient background on the role of general practitioners (GPs) in Denmark. To address this, we have now added the following statement to the Methods section: “In the Danish healthcare system, the general practitioner (GP) plays a central role for each patient, serving as the primary care provider, managing overall health, and coordinating care across sectors.” (line 149). This addition clarifies the integral role of GPs in the Danish context, which justifies their inclusion in the study, even though it was not explicitly mentioned in the original objectives. The involvement of GPs is essential for understanding the full spectrum of care provided to patients, especially those with complex health needs across sectors.*

==============================

Reviewers' comments:

**Comments to the Author**

**Reviewer #1:** Thank you very much for the opportunity to review this article. Overall, it is a very good piece and it is great to see more work is being done in this area. I have a few notes (please see below).

1. Introduction: you mention both mental illness and severe mental illness (SMI). SMI has been referred to as schizophrenia and bipolar, however, no examples have been given for mental illness – should all of this say severe mental illness? Also, more background could be given on what severe mental illness is. Such as what is schizophrenia/bipolar etc and how many people are affected by it? Are there any other severe mental illnesses?

2. Methods: It is not clear if the patients were diagnosed with a SMI or a mental illness. Many sources now recognise SMI as ‘bipolar, schizophrenia, and other psychoses’. There is a mixture of different illnesses within the patients with a lack of justification and links between the them.

*Points 1 and 2, please see the answer above about the definition of severe mental illness.*

3. Results subtheme 1 (line 295-316): It would be beneficial to see some more quotes here to justify the descriptions.

4. Results subtheme 2 (lines 320-334): It would be good to see a quote/quotes, specifically from the patients here.

5. Results subtheme 3 (lines 354-406): lacking any quotes to justify text.

*Points 3, 4 and 5, thank you for the opportunity to elaborate and further support our analysis. In response to your comments, we have added eight quotes to provide additional justification for our descriptions. Please see in the manuscript, Subtheme 1, 2 and 3.*

6. Strengths and limitations (line 556): ‘We chose not to investigate patients with ADHD or dementia, as they have very specialized trajectories that did not align with the aim of this study” – which trajectories are those and how do they differ from the trajectories of those you have included?

*Thank you for your question regarding the exclusion of patients with ADHD and dementia from our study. The central reason for this decision is that ADHD and dementia did not meet our criterion of prolonged and complex engagement with secondary psychiatric care, which was a key focus of our study. The reason is now specified in the Strengths and Limitations section. We hope this clarification addresses your concern.*

7. Conclusion (lines 561-563): very short, would be good to highlight the main recommendations you make here.

*We appreciate the reviewer’s suggestion to expand the conclusion. In response, we have revised the conclusion to offer a more detailed summary of our key findings and to clearly articulate the main recommendations stemming from our study.*

*“Conclusion
This study underscores the complexity of care for patients with cancer who also suffer from severe mental illness, revealing key areas for improvement. Our analysis identified the overarching theme of "Complexity on many levels," with four subthemes: the impact of the cancer trajectory on mental illness, the multifaceted vulnerability of patients, the fragmentation and lack of structure in the healthcare system, and the critical role of the patient-health professional relationship. Barriers to effective care included the exacerbation of mental illness due to the cancer trajectory and systemic fragmentation. Facilitators included recognizing and leveraging the patient's own resources and adopting a person-centered approach to care. By addressing these barriers and enhancing these facilitators, healthcare providers can better meet the needs of this vulnerable population.”*

Overall, a very interesting article, however, the article goes back and forth between mental illness and severe mental illness. I think this due to not having a solid explanation as to what severe mental illness is and its definition.

-----------------------------------------------------------

**Reviewer #2:** The author claims that he has done the study with a hermeneutic approach! Unfortunately this claim has not been confirmed in any part of the study.
The author has only conducted a qualitative content analysis. You can rewrite the study in the form of qualitative content analysis.

*We appreciate the concern regarding the use of the term "hermeneutic approach" in our manuscript. After careful consideration, we respectfully maintain that the study is conducted within a hermeneutic framework. To address the reviewer's concern and to clarify our approach, we have expanded the Methods section to provide a more detailed explanation of the hermeneutic principles guiding our analysis.
These revisions include additional background on the hermeneutic methodology and a more explicit description of how this approach was applied throughout the study. We believe these enhancements will make the hermeneutic nature of our analysis clearer.*

============================== END ==============================
